# A novel deep learning-based algorithm combining histopathological features with tissue areas to predict colorectal cancer survival from whole-slide images

**DOI:** 10.1186/s12967-023-04530-8

**Published:** 2023-10-17

**Authors:** Yan-Jun Li, Hsin-Hung Chou, Peng-Chan Lin, Meng-Ru Shen, Sun-Yuan Hsieh

**Affiliations:** 1 Institute of Medical Informatics, National Cheng Kung University, Tainan, 70101 Taiwan; 2 Department of Computer Science and Information Engineering, National Chi Nan University, Nantou, 545301 Taiwan; 3 Department of Oncology, National Cheng Kung University Hospital, College of Medicine, National Cheng Kung University, Tainan, 70101 Taiwan; 4 Department of Obstetrics and Gynecology, National Cheng Kung University Hospital, College of Medicine, National Cheng Kung University, Tainan, 70101 Taiwan; 5 Department of Computer Science and Information Engineering, National Cheng Kung University, Tainan, 70101 Taiwan

**Keywords:** Deep learning, Cancer survival, Tissue areas, Histopathological features, Tissue areas, Whole slide images

## Abstract

**Background:**

Many methodologies for selecting histopathological images, such as sample image patches or segment histology from regions of interest (ROIs) or whole-slide images (WSIs), have been utilized to develop survival models. With gigapixel WSIs exhibiting diverse histological appearances, obtaining clinically prognostic and explainable features remains challenging. Therefore, we propose a novel deep learning-based algorithm combining tissue areas with histopathological features to predict cancer survival.

**Methods:**

The Cancer Genome Atlas Colon Adenocarcinoma (TCGA-COAD) dataset was used in this investigation. A deep convolutional survival model (DeepConvSurv) extracted histopathological information from the image patches of nine different tissue types, including tumors, lymphocytes, stroma, and mucus. The tissue map of the WSIs was segmented using image processing techniques that involved localizing and quantifying the tissue region. Six survival models with the concordance index (C-index) were used as the evaluation metrics.

**Results:**

We extracted 128 histopathological features from four histological types and five tissue area features from WSIs to predict colorectal cancer survival. Our method performed better in six distinct survival models than the Whole Slide Histopathological Images Survival Analysis framework (WSISA), which adaptively sampled patches using K-means from WSIs. The best performance using histopathological features was 0.679 using LASSO-Cox. Compared to histopathological features alone, tissue area features increased the *C*-index by 2.5%. Based on histopathological features and tissue area features, our approach achieved performance of 0.704 with RIDGE-Cox.

**Conclusions:**

A deep learning-based algorithm combining histopathological features with tissue area proved clinically relevant and effective for predicting cancer survival.

**Supplementary Information:**

The online version contains supplementary material available at 10.1186/s12967-023-04530-8.

## Introduction

Evaluation of pathological images is considered the gold standard for cancer diagnosis and prognosis [1, 2]. Many pathological characteristics are useful in predicting the prognosis of colorectal carcinoma (CRC). Some of the histology cell features are important, such as the tumor characteristics, lymphocytes, stroma, and mucinous status on pathology images [3–6]. The features of tumor tissue, including the histology differential grade, endophytic tumor configuration pattern, and tumor budding, were correlated with tumor recurrence in patients with stage II–III CRC [3]. Stromal tissues with PD-L1-expressing immune cells have been reported to be associated with a favorable prognosis. In terms of histological segment features, the tumor-stroma ratio and tumor-lymphocyte infiltration have also been associated with prognosis [7, 8]. Although there are many prognostic factors on histology whole-slide images (WSIs), pathologists cannot quantify the characteristics of histology images and annotate the tissue regions related to patient outcomes. Many computational methods have been proposed to predict survival using pathological images [9]. Detecting and classifying cells on histopathological images would allow clinicians to predict patient outcomes, make precise decisions about therapies, and provide health care. However, obtaining clinically significant and explainable features from gigapixel WSIs with diverse tissue appearances remains challenging for an improved training model. Therefore, selecting image patches and segmenting tissues from WSIs to develop a survival prediction method are crucial.

Deep learning has been widely applied in pathological imaging tasks [10, 11]. Survival prediction can be divided into region-of-interest (ROI) and WSI-based methods. ROI-based methods typically sample patches from the tumor area labeled by pathologists and use neural networks to extract features from the patches for survival prediction [12–15]. Zhu et al. proposed a deep convolutional survival model (DeepConvSurv) to predict survival from pathological images [12]. Pathologists annotated image regions within each tumor as the ROIs and sampled patches from the ROIs as the input for the DeepConvSurv model. However, the annotation process could be more laborious and time-consuming for clinical applications. In addition, the model can only obtain tumor features and cannot quantify the characteristics of other tissues, such as lymphocytes and stroma, because of the limitations of the labeled region. Thus, WSI-based methods have attempted to capture various tissue features from WSIs.

WSI-based methods usually first sample patches from WSIs and select survival-related patches [16–20]. The models then extract features from the selected patches using neural networks and aggregate the features for survival prediction. For instance, Zhu et al. proposed a framework called the Whole Slide Histopathological Images Survival Analysis framework (WSISA) to predict survival using WSIs directly [16]. WSISA adaptively sampled patches from WSIs and used K-means to cluster the patches [21]. Each cluster was used to train the DeepConvSurv model [12]. Clusters with better predictive power than random guessing [concordance index (*C*-index) > 0.5] were selected for aggregation and prediction. Because gigapixel WSIs are too large to fit in the graphics processing unit (GPU), WSI-based methods use patches instead of WSIs to train deep learning models. However, extracting features from patches ignores the location and quantity of tissues and cannot capture clinically significant histopathological characteristics of WSIs. Recently, Li et al. used a graph convolutional neural network (GCN) to integrate spatial information from WSIs for survival prediction [22]. However, the spatial information of a few patches cannot be used to represent the location and quantity of tissues.

To address these problems, we propose a survival prediction method based on histopathological and tissue area features extracted from WSIs. The histopathological features were extracted from patches of actual tissue types (tumor, lymphocytes, stroma, and mucus) using the DeepConvSurv model, and the tissue area features were extracted from the tissue maps of WSIs by localizing and quantifying the tissue region (tumor, lymphocytes, and stroma) using image-processing techniques.

## Methods

### Data sources

This study used two public datasets from different patient cohorts: the National Center for Tumor Diseases (NCT)-Colorectal Cancer (CRC)-(hematoxylin and eosin) HE-100K [23] and the Cancer Genome Atlas Colon Adenocarcinoma (TCGA-COAD) databases. NCT-CRC-HE-100K consisted of patches sampled from slides, whereas TCGA-COAD contained WSIs. All images were stained with hematoxylin and eosin (HE) from formalin-fixed, paraffin-embedded (FFPE) samples. NCT-CRC-HE-100K comprises 100,000 patches sampled from 86 slides of human cancer tissue from the National Center for Tumor Diseases (NCT) biobank and the University Medical Center Mannheim (UMM) pathology archive (downloaded from http://dx.doi.org/10.5281/zenodo.1214456). All image patches are 224 × 224 pixels at 0.5 microns per pixel (MPP) and are color-normalized using Macenko’s method [24]. This dataset contains nine tissue classes: adipose (ADI), background (BACK), debris (DEB), lymphocytes (LYM), mucus (MUC), smooth muscle (MUS), normal colon mucosa (NORM), cancer-associated stroma (STR), and colorectal adenocarcinoma epithelium (TUM). NCT-CRC-HE-100K was used to train a ResNet50 classifier to identify the tissue type of an image patch and obtain the tissue map of the WSI.

We retrieved 258 WSIs from 252 colorectal cancer patients from The Cancer Genome Atlas Colon Adenocarcinoma (TCGA-COAD) (downloaded from https://portal.gdc.cancer.gov/) with survival data from the University of California, Santa Cruz (UCSC) using Xena (downloaded from https://xenabrowser.net/datapages/). We selected patients with WSIs and overall survival (OS) data. The dataset was used to evaluate the proposed method.

### Our proposed approach based on combinations of histopathological and tissue areas

**Fig. 1:**
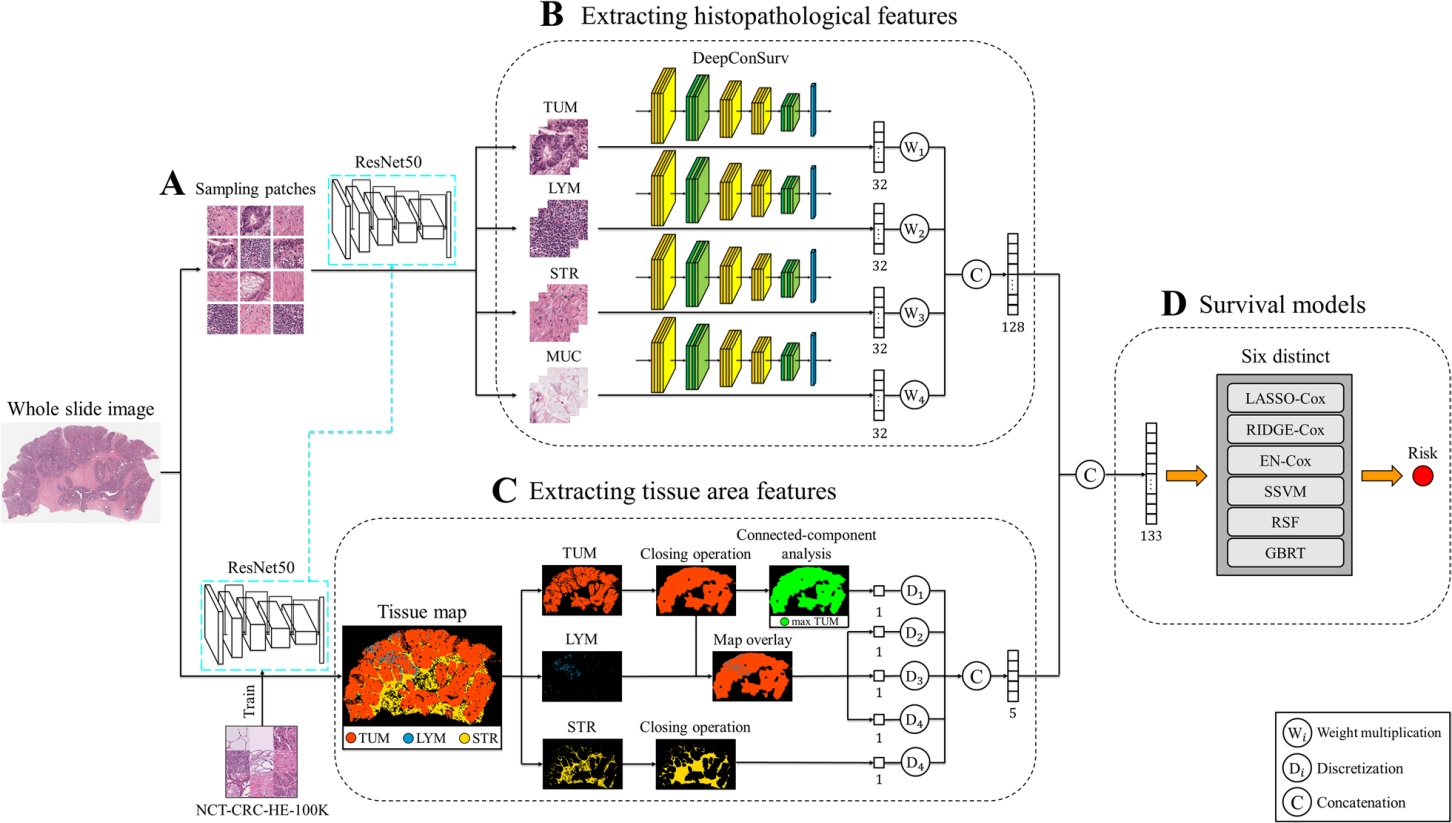
Overview of the proposed method. It aims to extract prognostic features from whole slide images and predicts patients’ survival risk. Our approach consists of four main parts: **A** sampling patches from whole slide images; **B** use of DeepConvSurv models to extract histopathological features of the tumor, lymphocyte, stroma, and mucus; **C** extraction of tissue area features from the tissue map by considering the tumor, lymphocyte, and stroma area; **D** training of several survival models using extracted features to predict patients’ risk. *LYM* lymphocyte, *MUC* mucus, *STR* stroma, *TUM* tumor, *SSVM* survival support vector machine, *RSF* random survival forest, *GBRT* gradient boosted regression tree

The proposed method comprised three main parts (Fig. 1). First, we extracted the histopathological features of tumors, lymphocytes, stroma, and mucus using DeepConvSurv models [12]. Second, tissue area features were retrieved from the tissue maps by evaluating the areas and ratios of the tumors, lymphocytes, and stroma. Third, we used extracted histopathological and tissue area features to predict patient risk using six survival models. An overview of the proposed method is presented in Fig. 1. We aimed to use WSIs with extracted prognostic features to forecast patient survival risk.

#### Patch sampling from whole-slide images

We randomly sampled patches with sizes of 224 × 224 pixels from the WSIs at 20X objective magnification. Since the WSIs are under the same magnification, the actual size of the tissue can be represented using the number of tissue patches. The sampling ratio was fixed according to the image size (here, we used 5%). The sampled patches were heterogeneous and could contain different types of information. We randomly sampled patches without pre-eliminating selected patches. The number of sampled patches was determined by multiplying the number of patches in the WSI by a fixed ratio. After sampling patches, we used the NCT-CRC-HE-100K dataset to train a patch-based ResNet50 tissue classifier and used the model to classify the sampled patches into different tissue types (ADI, BACK, DEB, LYM, MUC, MUS, NORM, STR, and TUM) (Fig. 1A) [25]. We eliminated the background patches and used the DeepConvSurv model to extract features from other tissue types.

#### Extracting histopathological features

**Fig. 2:**
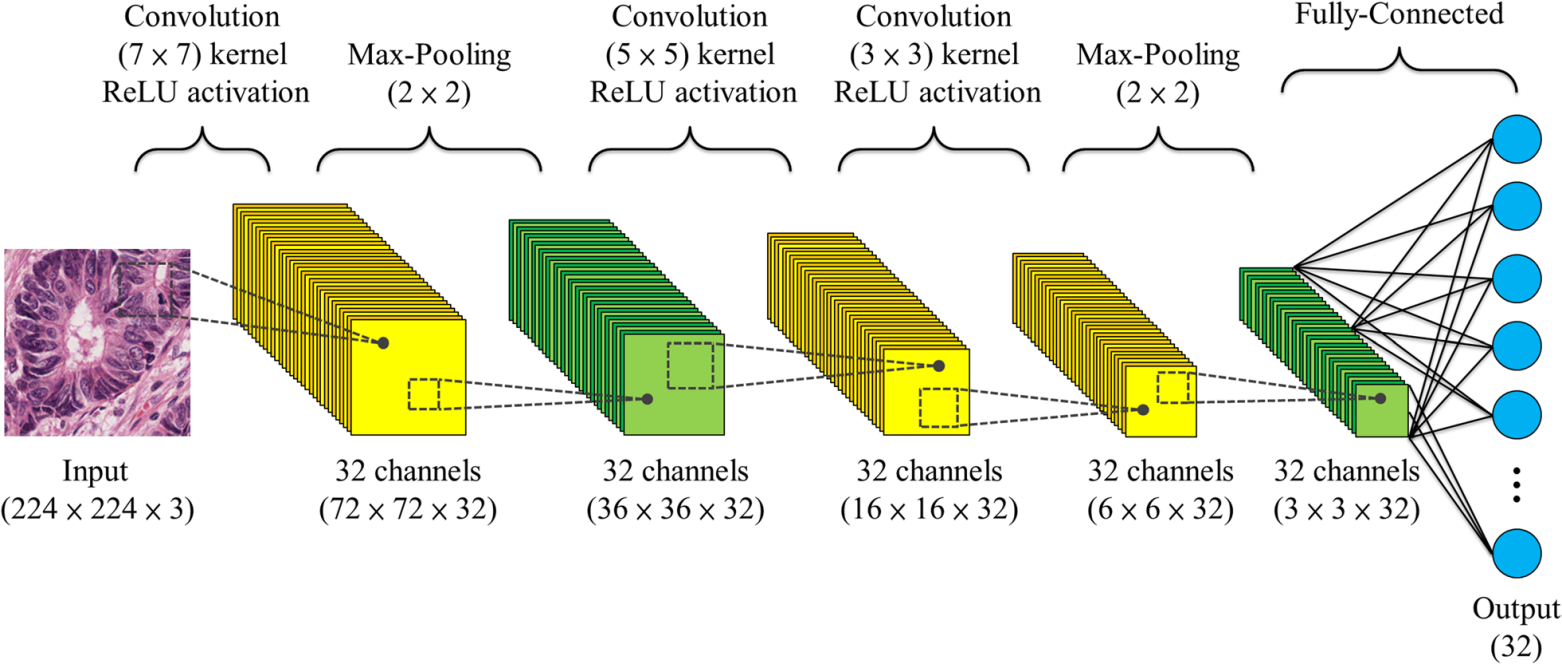
The architecture of the DeepConvSurv model. We trained the DeepConvSurv models separately on different tissue sets and used those models to extract histopathological features from patches

We first sampled patches from the WSIs and classified them into different tissue sets. Subsequently, we trained the DeepConvSurv models separately on different tissue types. The architecture of DeepConvSurv is shown in Figs. 1B and 2. DeepConvSurv extracts histopathological information from the image patches of nine different tissue types, including tumors, lymphocytes, stroma, and mucus [12]. The input of the DeepConvSurv model is 224 × 224 patches of different tissue types, and we extracted the last layer (a fully connected layer with 32 neurons) of the neural network and treated it as features. However, not all tissues are prognostic factors. In this paper, we used the combination of tumor, lymphocyte, stroma, and mucus, which achieved the best results, and these tissue types were also clinically significant (Fig. 1B). We combined these four tissue types (with 32 features) and thus obtained 4 × 32 = 128 histopathological features.

#### Extracting tissue area features from an image of a tissue map.

**Fig. 3:**
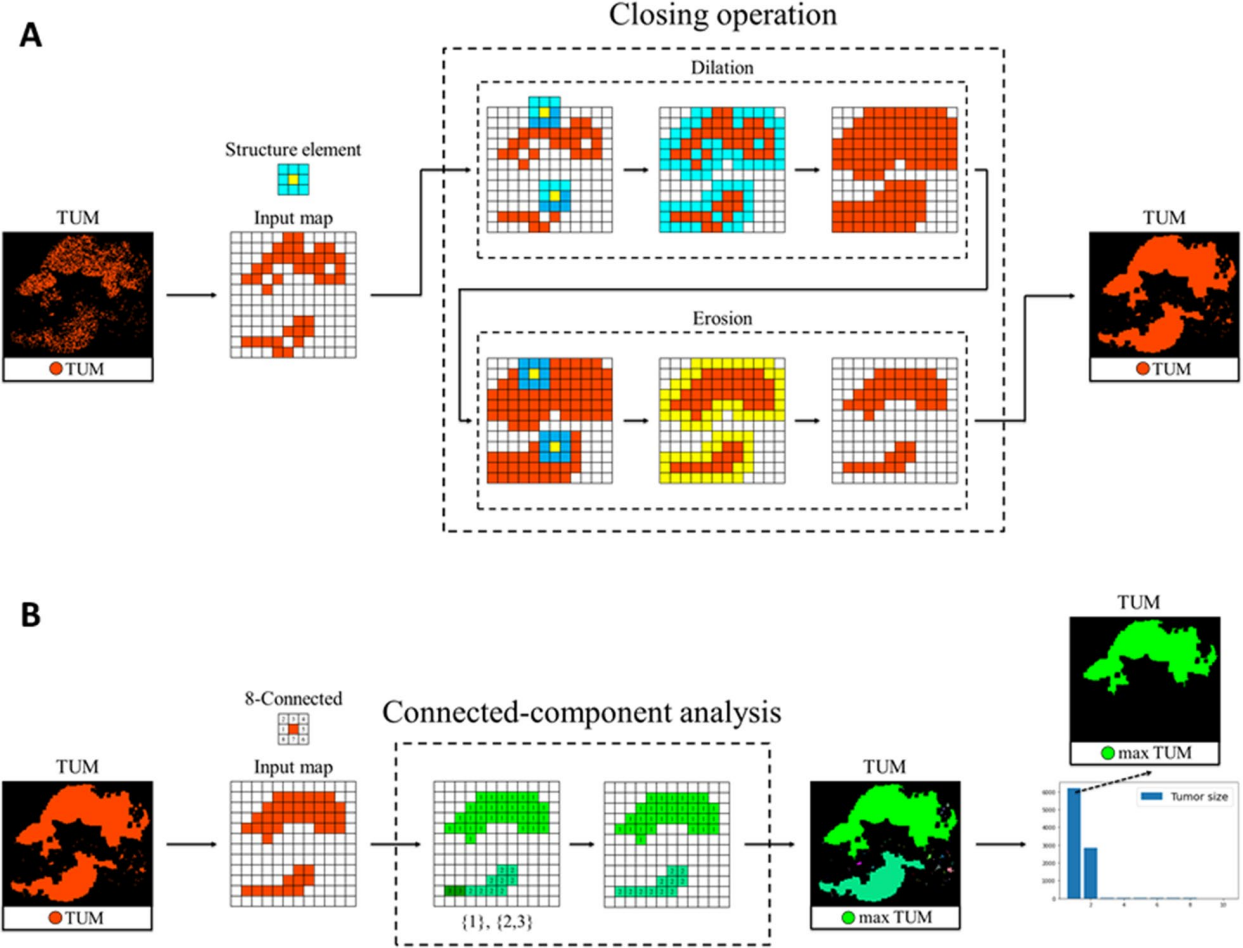
Image processing techniques for extracting tissue area features. We used the closing operation (**A**) to connect objects inappropriately divided into many small pieces. Connected-component analysis (**B**) was applied to find and label each connected component in an image. *TUM* tumor

We first cropped the WSI block by block into 224 × 224 patches to obtain the initial tissue map. Second, we used the ResNet50 classifier to classify these patches into tissues (tumor, lymphocytes, stroma, and others). The single patch label is determined by the proportion of the relatively large area of histological type. We then extracted features by considering the area of the tissues on the tissue map (tumor, lymphocytes, and stroma). Since tumor volume is one of the most important prognostic markers in cancer [26], it should be considered in survival models. In addition to tumors, we pondered whether the volumes of lymphocytes and stroma should also be considered. Hence, we localized and quantified the regions of these three tissues in the WSIs to extract features. WSIs were cropped into patches, and we used the pretrained ResNet50 classifier to classify the patches into four classes (tumor, lymphocytes, stroma, and others) [25]. Third, we use the classification results to map different colors to patches of various tissues and then obtained a tissue map. The tissue map of the WSIs was segmented using image-processing techniques that involved localizing and quantifying the tissue region. We used several image-processing techniques (Figs. 1B and 3) to extract the tissue area features, including closing operations and connected-component analysis. The closing operation was dilation, followed by erosion. Dilation connects objects inappropriately divided into many small pieces, making the objects larger. Therefore, erosion shrinks the objects that are used. The connected-component analysis labels each component so that we can capture our interested components.

For tumor patches on the tissue map, we first used the closing operation, a structuring element of a 5 × 5 rectangle, to reinforce the patch classification results [27]. It was used to connect objects that were inappropriately divided into many small pieces to obtain tumors. Second, we applied connected-component analysis [28] to capture the maximal tumor and calculate its area (max_tumor_area) (here, we considered eight connected components). For lymphocyte patches on the tissue map, we calculated the area around and inside the tumors (lymphocyte_inside_tumor, lymphocyte_around_tumor) because they affect prognosis differently. To combine the power of these two features, we calculated their ratio (around_inside_ratio). To address a zero number of lymphocyte patches, we added 1 to the numerator and denominator. For stroma patches on the tissue map, we also used the closing operation (here, we used a structuring element of a 5 × 5 rectangle), and then the total area (total_stroma_area) was calculated. The five features mentioned above are called tissue area features. Except for the internal ratio, the unit was the number of patches.

#### Survival models and metrics evaluation

To assess the prognostic power of the extracted features, we trained six different survival models, including statistical methods (least absolute shrinkage and selection operator (LASSO)-Cox [29], RIDGE-Cox [30], elastic net (EN)-Cox [31]), survival support vector machine (SSVM) [32], random survival forest (RSF) [33], and gradient boosted regression tree (GBRT) [34]). In this study, overall survival (OS) data were used as the outcome measure. Fivefold cross-validation was used to obtain a reliable result (Fig. 1D).

The concordance index (*C*-index) was used as the evaluation metric. The concept of the *C*-index is that patients at higher risk should have shorter survival times. The *C*-index measures the concordant pairs between survival time and prediction risk and is computed as follows:

1 $$ C{\text{ - index }} = \frac{{\mathop \sum \nolimits_{i \in U} \mathop \sum \nolimits_{{T_{j} > T_{i} }} \mathop \sum \nolimits_{{R_{i} > R_{j} }} 1}}{{\mathop \sum \nolimits_{i \in U} \mathop \sum \nolimits_{{T_{j} > T_{i} }} 1}} $$

where *U* is the set of uncensored data, *T* is the observed time, and *R* is the predicted risk. The *C*-index ranges from 0 to 1, with higher values indicating better performance. A *C*-index value of 1 indicates perfect prediction, and a *C*-index of 0.5 indicates a random guess.

### Implementation details

ResNet50 [25] and DeepConvSurv models [12] were constructed using the *PyTorch* package (version 1.8.1, accessed on May 11, 2021) in Python. We used the Adam optimizer to train the models on a single NVIDIA GeForce RTX 2080 GPU with 8 GB of memory. ResNet50 was pretrained using the ImageNet dataset [35] and employed a cross-entropy loss function. The DeepConvSurv models were initialized using the He method [36], and the negative log partial likelihood loss function was used. All comparison survival models (LASSO-Cox, RIDGE-Cox, EN-Cox, SSVM, RSF, and GBRT) were built using the scikit-survival package (version 0.14.0, accessed on June 7, 2021) in Python. The maximally selected rank statistics method [37] from the survminer package (version 0.4.9, accessed on October 15, 2021) and the Kaplan‒Meier method from the *survival* package (version 3.2–13, accessed on October 15, 2021) were implemented in R. The source code is publicly available at https://github.com/v1x99y7/WSI-HSfeatures.

## Results

### Identification of histopathological features based on DeepConvSurv models

We assigned labels to each patch using the patients’ overall survival (OS) data and used pretrained DeepConvSurv models to extract features from tissues. However, not all tissues are prognostic factors. To determine which combination of tissues correlated the most with survival, we trained six survival models (LASSO-Cox, RIDGE-Cox, EN-Cox, SSVM, RSF, and GBRT) for all combinations of tissues except the background (total 2^8^–1 = 255 combinations). The combination of tumor, lymphocytes, stroma, and mucus achieved the best results (Table 1), and these tissue types were also clinically significant. Other tissues, such as adipose, debris, muscle, and normal mucosa, were less correlated with survival. Therefore, we selected four tissue types for this study.

**Table 1:** Performance of the histopathological features via fivefold cross-validation using *C*-index values

Method	TUM^a^	LYM^a^	STR^a^	MUC^a^	TUM + LYM + STR + MUC^a^
LASSO-Cox	0.521 ± 0.041	0.448 ± 0.054	**0.566 ± 0.042**	0.559 ± 0.065	**0.687 ± 0.084**
RIDGE-Cox	0.615 ± 0.059	**0.567 ± 0.068**	0.532 ± 0.107	0.589 ± 0.058	0.616 ± 0.092
EN-Cox	0.546 ± 0.036	0.443 ± 0.065	0.539 ± 0.037	0.573 ± 0.079	0.646 ± 0.064
SSVM	**0.616 ± 0.042**	0.565 ± 0.069	0.479 ± 0.085	**0.596 ± 0.088**	0.598 ± 0.095
RSF	0.601 ± 0.057	0.455 ± 0.065	0.498 ± 0.111	0.556 ± 0.107	0.605 ± 0.078
GBRT	0.551 ± 0.087	0.443 ± 0.036	0.536 ± 0.062	0.560 ± 0.058	0.610 ± 0.052

The results highlighted in bold black show the best performance with those methods

^a^*LYM* lymphocyte, *MUC* mucus, *STR* stroma, *TUM* tumor

We treated the output of the fully connected layer in the DeepConvSurv model as a feature. For each patch, we obtained a 32-dimensional feature. The features of each tissue type are obtained by averaging the features of the patches in this tissue set and multiplying them by a weight. The weight is the percentage of the number of patches in the tissue out of the number of all patches. Finally, we concatenated the four 32-dimensional features of tissue types to obtain an overall 128-dimensional feature.

### Identification of tissue area features

**Fig. 4:**
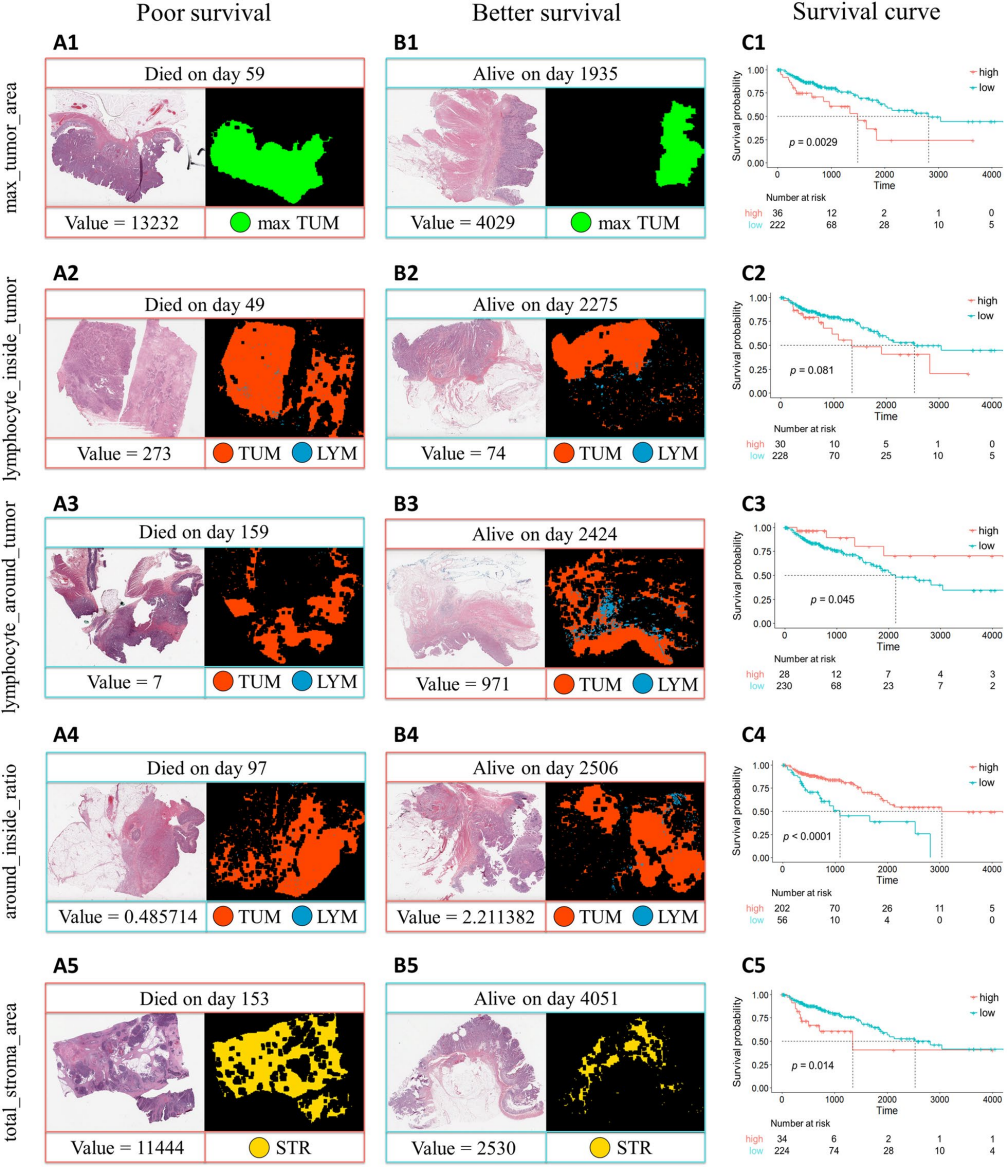
Examples of poor (**A1**–**A5**), and better survival cases (**B1**–**B5**) and corresponding Kaplan‒Meier survival curves (**C1**–**C5**) of tissue area features including max_tumor_area, lymphocyte_inside_tumor, lymphocyte_around_tumor, around_inside_ratio, and total_stroma_area. The cutoff point to categorize tissue area features as high-group and low-group were determined by the maximally selected rank statistics method. There is a whole slide image (left) and a visualized map (right) in each case (**A1**–**A5** and **B1**–**B5**). The red outer frame indicates a high-group case (**A1**, **A2**, **B3**, **B4**, and **A5**), and the blue outer frame represents a low-group case (**B1**, **B2**, **A3**, **A4**, and **B5**). *LYM* lymphocyte, *STR* stroma, *TUM* tumor

We extracted five clinically prognostically relevant and explainable tissue area features, including max_tumor_area (*p* value = 0.0029), lymphocyte_inside_tumor (*p* value = 0.081), lymphocyte_around_tumor (*p* value = 0.045), around_inside_ratio (*p* value < 0.0001), and total_stroma_area (*p* value = 0.014). Details of the tissue area features are listed in Table 2. To understand the prognostic power of tissue area features, we first determined the cutoff points of tissue area features using maximally selected rank statistics [37] and partitioned the patients into two groups to compute survival curves using the Kaplan‒Meier method. The survival curves are presented in Fig. 4 (C1–C5). The log-rank test was used to compare the survival distributions of the different groups. We observed that the tissue area features had significant impacts on survival.

**Table 2:** Details of the tissue area features

Tissue area feature	Definition	Cutoff point^a^	*p* value**
max_tumor_area	The area of max tumor	11,854	0.0029
lymphocyte_inside_tumor	The area of lymphocytes inside tumors	245	0.081
lymphocyte_around_tumor	The area of lymphocytes around tumors	388	0.045
around_inside_ratio	lymphocyte_around_tumor + 1/lymphocyte_inside_tumor + 1	0.8581315	< 0.0001
total_stroma_area	The area of total stroma	7324	0.014

^a^The cutoff point is determined by the maximally selected rank statistics method

^**^ *p* value is determined by the log-rank test

Although the *p* value of lymphocyte_inside_tumor was not less than 0.05 (*p* value = 0.081), we still considered it because it has a different impact from lymphocyte_around_tumor. By calculating their ratio, we obtained a statistically significant feature (around_inside_ratio, *p* value < 0.0001). Because patients in different groups have different survival situations, we discretized the tissue area features (if the value of the feature was greater than the cutoff point, we set it to 1; otherwise, we set it to 0) and concatenated them with histopathological features.

### Case studies of survival analysis based on tissue area features

Figure 4A1–A5 shows poor survival cases, and Fig. 4B1–B5 shows better survival cases. The corresponding Kaplan‒Meier survival curves (C1–C5) of tissue area features, including max_tumor_area, lymphocyte_inside_tumor, lymphocyte_around_tumor, around_inside_ratio, and total_stroma_area, were determined by the maximally selected rank statistics method. Max_tumor_area, lymphocyte_inside_tumor, and total_stroma_area were associated with poor survival. Lmphocyte_around_tumor and around_inside_ratio were associated with better survival.

### Cancer survival prediction based on histopathological features and tissue areas

By merging 128 histopathological features and five tissue area features, we obtained a 133-dimensional feature and assessed its prognostic power using six distinct survival models. By concatenating histopathological and tissue area features, we obtained the final 133 features. To evaluate the performance of the proposed method, we used six survival models: LASSO-Cox, RIDGE-Cox, EN-Cox, SSVM, RSF, and GBRT. The Cox model is a semiparametric model most commonly used for survival analysis. We used the l1-norm (LASSO-Cox), l2-norm (RIDGE-Cox), and elastic net penalized Cox (EN-Cox) models. The SSVM uses a kernel trick to obtain the nonlinear relationship between the features and survival. The RSF is an ensemble model that improves performance by averaging the predictions of the survival trees. The GBRT combines the predictions of multiple base regression trees with greedy addition. We compared the proposed method with WSISA, a state-of-the-art WSI-based method for survival prediction. We also compared the performance of histopathological features to further understand the predictive power of histopathological features and the ability of tissue area features to improve their performance. Table 3 shows the performance comparison via fivefold cross-validation with k-means.

**Table 3:** Performance comparison via fivefold cross-validation with K-means using the *C*-index value

Method	WSISA	Histopathological features	Histopathological + tissue area features
LASSO-Cox	0.556 ± 0.073	**0.679 ± 0.095**	0.694 ± 0.095
RIDGE-Cox	**0.620 ± 0.054**	0.656 ± 0.027	**0.704 ± 0.028**
EN-Cox	0.612 ± 0.032	0.651 ± 0.050	0.683 ± 0.043
SSVM	0.603 ± 0.075	0.657 ± 0.048	0.685 ± 0.037
RSF	0.504 ± 0.053	0.615 ± 0.074	0.651 ± 0.046
GBRT	0.498 ± 0.064	0.614 ± 0.036	0.621 ± 0.057

The results highlighted in bold black show the best performance with those methods

Our proposed method achieved better performance than WSISA in various survival models. Even with only histopathological features, we can obtain a better result than with WSISA. The best performance using histopathological features was 0.679 using LASSO-Cox. Compared with the best performance of WSISA using RIDGE-Cox, our method significantly improved the *C*-index by 5.9%. By combining histopathological features with tissue area features, our proposed method achieved performance of 0.704 using RIDGE-Cox. Using tissue area features improved the *C*-index by 2.5% compared with using histopathological features only.

## Discussion

Our results highlight the following important points. (i) a total of 128 histopathological features were extracted from four histological types and five tissue area features from WSIs to predict colorectal cancer survival; (ii) our method performed better in six distinct survival models than the WSISA adaptively sampled patches using K-means from WSIs; and (iii) using a novel deep learning-based algorithm combining tissue areas with histopathological features, we demonstrated a clinically relevant survival prediction model.

We extracted histopathological features from selected tissue sets, whereas WSISA extracted features from K-means clusters with better predictive power than random guessing (*C*-index > 0.5). We observed that the selected K-means clusters might contain prognostically small patches due to the selection strategy, which could have adversely affected the survival predictions. The results showed that selecting a specific tissue set considering expert advice and model performance can better extract prognostically significant patches and predict patient survival. However, extracting histopathological information from patches can only obtain histological cell features. To capture the histology segment features from the WSIs, we extracted tissue area features from the tissue map. The results showed that tissue area features could enhance the prediction performance of the histopathological features.

From the known literature, we selected six popular models [29–34], including three statistical methods and three machine learning methods. The six survival models manage features differently to better assess the prognostic power of the extracted features. Statistical methods can address linear relationships between features, while machine learning methods can obtain nonlinear relationships. The localization and quantification of WSI features provide a more objective method to evaluate slides. We located and quantified tissues using tissue area features that are prognostic and explainable. The survival curve of max_tumor_area (Fig. 4C1) showed that larger tumors led to poorer survival, consistent with the known tumor volume biomarker. We observed that not all lymphocytes had the same effects on survival. More lymphocytes inside tumors led to poorer survival (Fig. 4C2), whereas more lymphocytes around tumors led to better survival (Fig. 4C3). By calculating the ratio of these two features, we identified an influential prognostic factor, around_inside_ratio (*p* value < 0.0001) (Fig. 4C4). The survival curve of total_stroma_area (Fig. 4C5) showed that more stroma leads to poorer survival. These factors could assist pathologists in making diagnoses. In our study, we used deep learning and image processing techniques to capture the areas of different tissues and considered them significant features. For example, tumor size is an important biomarker that is clinically relevant. We showed that these area features are prognostic and explainable.

In this study, we used eight clinical features, including adipose (ADI), debris (DEB), lymphocytes (LYM), mucus (MUC), smooth muscle (MUS), normal colon mucosa (NORM), cancer-associated stroma (STR), and colorectal adenocarcinoma epithelium (TUM). To determine which combination of tissues was most correlated with survival, six survival models were trained (LASSO-Cox, RIDGE-Cox, EN-Cox, SSVM, RSF, and GBRT). TUM, LYM, STR, and MUC were the most effective combinations (Table 1). Conversely, adipose tissue, debris, muscle, and normal mucosa were less correlated with survival (Additional file 1: Table S1). Many pathological characteristics can be used to predict the prognosis of colorectal carcinoma (CRC), including tumor characteristics, lymphocytes, stroma, and mucin content [3–6]. Compatible with the clinical pathological findings, our selected four tissue types were also significant. We extracted 128 histopathological features from four histological types.

In recent studies, tumor-lymphocyte infiltration and the tumor-stroma ratio were also related to prognosis [7, 8]. In addition, we found that max_tumor_area, lymphocyte_inside_tumor, lymphocyte_around_tumor, around_inside_ratio, and total_stroma_area were related to cancer survival. For example, lymphocyte_inside_tumor, lymphocyte_around_tumor, around_inside_ratio, and total_stroma_area were associated with the tumor microenvironment. There are some studies that show that fat invasion of colorectal tumors is a prognostic factor [38, 39]. However, adipose tissue was less correlated with survival (Additional file 1: Table S1) in this study. We did not conduct the fat invasion of colorectal tumors study. In the future, we may focus on cancer-associated adipocyte or peritumoral fat invasion by computational pathology. This study quantified and extracted five tissue area features from whole slide images (WSIs). Finally, we added the five tissue area features to the 128 histopathological features from four histological types to predict cancer survival. The study aims to develop a computational pathology approach to extract tissue area features. We use public datasets that were not annotated by pathologists. Our results were not compared with annotations by pathologists.

DeepConvSurv was used to extract histopathological features in this study. If we apply novel models, such as Transformers [40], CLAM, or Streaming, we might overcome the limitations of a patch-based method and improve the prediction performance. For example, transformers [40] have been shown to improve the results of many tasks with the help of the attention mechanism. They require a large dataset or pretrained weights to train the models. However, we require the same model (DeepConvSurv model) used in the previous WSISA study to demonstrate the validity and importance of tissue area features. To obtain clinically significant and explainable features of tissue areas, we compared the performance among WSISA, histopathological features only, and histopathological plus tissue area features (Table 3). The performance improvement compared to WSISA with the same model was due to the power of tissue area features, rather than the model itself.

The ResNet50 tissue classifier has overall accuracy of 93%. For the tissue area, we used the closing operation, consisting of dilation and erosion, to reinforce the classification results by connecting objects that were inappropriately divided into many small pieces, which might have improved the segmentation performance. Pathologists’ annotations are time-consuming and labor-intensive for tumors, lymphocytes, stroma, and mucus, so the study aimed to quantify and extract prognostic features from WSIs without pathologists' labels. This study used the concordance index (C-index) as the evaluation metric. The C-index measures concordant pairs among patient pairs by comparing two patients’ survival times and prediction risks. A pair is concordant if the patient with the higher risk has a shorter survival time. Since the C-index evaluates performance from an overall patient perspective, we were not able to select an individual case in which the method did not predict well [41].

For digital pathology, stain-normalization is important, especially in patches [42, 43]. The trained datasets are normalization datasets in our study. However, the gigapixel whole slide images (WSIs) are too large to normalize. Normalization of the resection is also important. The normalization by the ratio of tumor patches is another method to make meaningful insights. We did not use the ratio of tumor patches. In the study, we use the whole slide image with the same 20X magnification. In clinical practice, actual tumor sizes were correlated with survival [3]. By comparing the tumor patches at the same magnification, we can calculate the exact size of the tumor.

There are several limitations of this study. First, the size of the TCGA-COAD dataset is limited. More datasets should be used to validate the generalizations of the method. Second, the patch sampling rate was 5%, which might have caused some patches containing important information to not be sampled. For a gigapixel WSI, tens of thousands of patches could result in excessive training time. More efficient ways to sample significant patches should be further explored. Third, some new WSI-based survival prediction methods have recently been proposed and have performed well. These studies should also be used for comparison with the proposed method. Fourth, the patient may have serial pathology slides in clinical practice. In this study, our model is compared with the WSISA model [16]. In the WSISA study, the number of WSIs and patients differed [16]. One patient has two slides. Therefore, we apply the same overall survival label to a case with two slides. However, this might introduce some noise into the results.

## Conclusions

We have proposed an approach for selecting histopathological images using deep-learning-based histopathological features and tissue area from WSIs to predict cancer survival. Our method outperformed WSISA K-means sampling patches in six distinct survival models. In addition, we have provided clinically relevant and explainable features by tissue areas. In the future, we will investigate more ways to extract clinical prognostic features from WSIs and build survival prediction models.

### Supplementary Information

**Additional file 1: Table S1.** The performance of different combinations of histopathological tissue.

## Data Availability

The code used for the analysis in this paper is available online at https://github.com/v1x99y7/WSI-HSfeatures. Any additional information required to reanalyze the data reported in this paper is available from the lead contact upon request.

## Abbreviations

ADI: Adipose
BACK: Background
C-index: Concordance index
CRC: Colorectal carcinoma
DEB: Debris
DeepConvSurv: Deep convolutional survival model
FFPE: Formalin-fixed, paraffin-embedded
GBRT: Gradient boosted regression tree
GCN: Graph convolutional neural network
GPU: Graphics processing unit
HE: Hematoxylin and eosin
LASSO: Least absolute shrinkage and selection operator
LYM: Lymphocytes
MPP: Microns per pixel
MUC: Mucus
MUS: Smooth muscle
NCT: National center for tumor diseases
NORM: Normal colon mucosa
OS: Overall survival
ROI: Regions of interest
RSF: Random survival forest
SSVM: Survival support vector machine
STR: Cancer-associated stroma
TCGA-COAD: The cancer genome atlas colon adenocarcinoma
TUM: Colorectal adenocarcinoma epithelium
UCSC: University of California, Santa Cruz
UMM: University Medical Center Mannheim
WSIs: Whole-slide images
WSISA: Whole slide histopathological images survival analysis framework
